# The Interplay between Oxidative Stress and Fatty Acids Profile in Romanian Spotted Cows with Placental Retention

**DOI:** 10.3390/vetsci11100499

**Published:** 2024-10-12

**Authors:** Sanda Andrei, Horațiu Rafa, Ioan Oroian, Oana Maria Cozma, Andreea Georgiana Morohoschi, Daria Antonia Dumitraș, Francisc Dulf, Cristina Laura Ștefănuț

**Affiliations:** 1 Department of Preclinical Sciences, Faculty of Veterinary Medicine, University of Agricultural Sciences and Veterinary Medicine Cluj-Napoca, 400372 Cluj-Napoca, Romania; horatiu.rafa@usamvcluj.ro (H.R.); andreea-georgiana.morohoschi@student.usamvcluj.ro (A.G.M.); antonia.dumitras@usamvcluj.ro (D.A.D.); cristina.stefanut@usamvcluj.ro (C.L.Ș.); 2 Stațiunea de Cercetare—Dezvoltare pentru Creșterea Bovinelor Târgu Mureș, 547430 Sângeorgiu de Mureş, Romania; scdbtgmures@yahoo.com; 3 Department of Clinical Sciences, Faculty of Veterinary Medicine, University of Agricultural Sciences and Veterinary Medicine Cluj-Napoca, 400372 Cluj-Napoca, Romania; popoanamaria1@gmail.com; 4 Department of Environmental Protection, Faculty of Agriculture, University of Agricultural Sciences and Veterinary Medicine Cluj-Napoca, 400372 Cluj-Napoca, Romania; francisc.dulf@usamvcluj.ro

**Keywords:** cow, parturition, placenta, CAT, SOD, MDA, TAC, fatty acids profile

## Abstract

**Simple Summary:**

Retained fetal membranes (RFM) are a common reproductive issue in cattle, affecting overall health and reproductive efficiency. This study examined oxidative stress (OS) and fatty acid profiles in Romanian Spotted cows, comparing those with normal calving to cows with RFM. Serum samples were collected from 22 cows over a 9-week period surrounding calving to measure key OS markers, including superoxide dismutase (SOD), catalase (CAT), malondialdehyde (MDA), and total antioxidant capacity (TAC). Placental tissues were also analyzed for OS markers and fatty acid composition. The results showed significant changes in OS before and after birth, with cows experiencing RFM exhibiting lower antioxidant activity and higher lipid damage. Additionally, RFM cows had higher saturated fatty acid levels in the placenta, indicating metabolic stress. These findings highlight the role of OS and fatty acid imbalances in RFM, suggesting potential ways to improve dairy cow health by addressing these factors.

**Abstract:**

(1) Background: Retained fetal membranes (RFM) in cattle negatively impact reproduction, calving intervals, and health. This study examined OS markers and fatty acid profiles in Romanian Spotted cattle, comparing cows with normal parturition to those with RFM. Over 9 weeks, serum samples were collected from 22 cows (7 with RFM, 15 normal) at intervals before and after parturition. Placental tissues were also analyzed. The aim was to identify OS biomarkers that predict RFMs, track changes over time, and assess their impact on the placental fatty acid profile. (2) Methods: Samples were analyzed for superoxide dismutase (SOD), catalase (CAT), malondialdehyde (MDA), and total antioxidant capacity (TAC). Placental fatty acids were profiled using gas chromatography–mass spectrometry. (3) Results: SOD and CAT activities increased in cows with retained fetal membranes (RFM) before parturition (SOD: *p* < 0.001, RFM 404.601 ± 20.941 vs. NP 339.101 ± 44.911; CAT: *p* < 0.01, RFM 121.132 ± 14.831 vs. NP 96.070 ± 2.397), indicating OS. However, significant decreases during labor suggested weakened antioxidant defenses. Total antioxidant capacity (TAC) peaked during parturition in RFM cows (*p* < 0.0001, 38.780 ± 3.727 vs. 11.150 ± 1.555), signaling heightened stress. Additionally, MDA levels increased before parturition (*p* < 0.001, RFM 8.424 ± 1.894 vs. NP 3.807 ± 0.484), confirming lipid peroxidation. RFM cows also exhibited higher levels of saturated fatty acids and lower monounsaturated fatty acids, pointing to metabolic stress. (4) Conclusions: This study highlights the role of OS and fatty acid imbalances in RFMs, suggesting potential strategies to improve reproductive outcomes by managing OS.

## 1. Introduction

The periparturient period in lactating cows, commencing approximately 3–4 weeks prior to parturition and spanning a total duration of approximately 6–8 weeks, is marked by substantial metabolic stress [1]. During this transitional phase, cows undergo intricate physiological changes to support the impending birth and lactation. However, this critical period is not without its challenges, as it is often associated with a spectrum of pathologies [2]. And among these pathophysiological conditions, incomplete placental expulsion, known as placental retention or retained fetal membranes (RFM), emerges as a concerning issue in bovine parturition [3]. An RFM primarily results from the improper detachment of fetal cotyledon villi from the maternal crypts within the uterine caruncle. While the standard process of placental separation and expulsion typically occurs within a time frame of 0.5 to 12 h, variations are observed based on factors such as cow parity and age [3]. Factors associated with RFM encompass preterm delivery, infectious processes, surgical interventions, pharmacological induction of labor, multiple gestations, immature placentomes, noninflammatory edema of the chorionic villi that results from uterine torsion and cesarean sections, dystocia, necrosis between the crypts and villi following a possible antepartum allergic reaction, premature involution of the placentomes, hyperemia of the placentomes, inflammation, mechanical prevention of expulsion, inflammation of fetal membrane and indirect factors like intensive stress, duration of pregnancy, extensive distension, season, sex of the fetus, stillbirth, deficiency of trace minerals and vitamins, and situations where the uterus becomes atonic and unable to contract in cases such as dropsy, twinning, fetal gigantism, and subclinical hypocalcemia [2,3]. The retention of fetal membranes is a pathological condition of significant concern, with important consequences for a cow’s future reproductive performance, as it impacts key factors such as the calving interval, the time from conception to calving, and the success rate of initial insemination [2]. Retained fetal membranes have been associated with prolonged abnormal vaginal discharge, delayed uterine involution, extended intervals until the first ovulation, and increased rates of endometritis [4].

Economically, RFM imposes a significant burden on the dairy industry, leading to considerable financial losses [2]. Based on the research conducted by Suthar et al. [5], and reviewed by Mordak et al. [6], in the European Union, RFM is estimated to affect around 10% of milk-producing cows, making it the third most prevalent condition on farms, following mastitis and milk fever.

Risk factors for retained placenta differ across regions due to variations in management practices, environmental conditions, and herd health strategies. A study conducted by Rafa et al. [7] on the same farm in Mureș County, Romania, evaluated factors such as breed, age, season, and calf sex concerning retained placenta. The findings revealed that breed had a significant impact on the incidence of retained placenta, with Balțata Românească cows (15.73%) being more affected than Pinzgau cows (8.33%).

Amidst the intricate interplay of pathophysiological factors contributing to RFM, emerging research has begun to shed light on the role of OS in this condition. Oxidative stress, a state characterized by an imbalance between the production of reactive oxygen species (ROS) and the capacity of antioxidant defenses, is a fundamental phenomenon in biology. It is marked by an increase in the concentration of compounds resulting from the oxidative degradation of biomolecules, including lipid peroxidation products such as 4-hydroxy-trans-2-nonenal, malondialdehyde, and glyoxal [8].

Cows with RFM exhibit elevated oxidative markers, particularly around the seventh postnatal day, suggesting a potential link between OS and placental retention [9]. Oxidative stress has the capacity to induce damage to critical intracellular structures, including DNA, within placental regions, further implicating its role in the pathogenesis of placental retention [10].

Moreover, OS is not unique to placental retention but is also associated with a spectrum of peripartum conditions in cattle, including mastitis, acidosis, ketosis, and pneumonia [11]. Within the antioxidant defense system, the enzymatic activities of superoxide dismutase (SOD) and catalase (CAT) hold significant importance in the regulation of ROS levels [12]. The synergistic action of these enzymes is essential for preventing the formation of the highly deleterious hydroxyl radical (HO·) [8].

The successful parturition of dairy cows is crucial for the sustainability and profitability of dairy farming. Complications like RFM can lead to severe economic losses due to increased labor, veterinary care costs, decreased milk production, and reproductive performance. Oxidative stress has been implicated as a potential contributing factor to RFM, suggesting that an imbalance between prooxidants and antioxidants may play a pivotal role. Investigating the fatty acid profile and the ratio of saturated to unsaturated fatty acids in relation to malondialdehyde (MDA) levels in the placenta can offer insights into the biochemical and OS factors associated with RFM. However, research into the OS profiles of specific dairy cow breeds, such as the Romanian Spotted cow, is sparse. This gap highlights a critical need for breed-specific studies to better understand and manage RFM in diverse genetic populations.

This study aims to explore the oxidative stress indicators and the fatty acids profile of the placenta associated with retained fetal membranes in Romanian Spotted cows, a breed of significant agricultural value yet underrepresented in scientific research. By analyzing key OS markers, namely CAT, SOD malondialdehyde (MDA), and total antioxidant capacity (TAC), in blood samples collected before, during, and after parturition, as well as in the fetal placenta and its fatty acid profile, this research aims to identify biomarkers that could predict or indicate RFM risk in Romanian Spotted cows; understand the dynamics of OS in relation to parturition and RFM; and evaluate how RFM impacts OS in the placenta, as reflected by placental markers and fatty acid profiles. Focusing on the Romanian Spotted cow breed provides novel insights into the breed-specific physiological and biochemical responses to parturition and RFM. This information can help tailor management and breeding strategies to reduce the incidence of RFM in this particular breed.

The longitudinal design of this study, tracking OS markers before, during, and after parturition, provides a detailed temporal profile, helping to pinpoint critical periods for effective interventions against retained fetal membranes (RFM). By analyzing oxidative damage indicators, such as malondialdehyde (MDA), antioxidants (CAT, SOD, TAC), and fatty acid profiles, the study offers a comprehensive view of the biochemical factors associated with RFM. This integrated approach uncovers complex interactions between OS pathways, fatty acid profiles, and RFM occurrence. Not only does the study fill a critical knowledge gap regarding RFM in Romanian Spotted cows, it also sets a foundation for future research on OS, fatty acids, and reproductive health in dairy cattle. The findings have potential implications for improving animal welfare, farm productivity, and economic outcomes. Additionally, as one of the first studies to focus on the Romanian Spotted breed, it provides valuable breed-specific data to guide targeted interventions and management practices. 

## 2. Materials and Methods

### 2.1. Chemicals and Reagents

The kits utilized in the current study were procured from Elabscience Biotechnology Inc., located in Houston, TX, USA. The additional chemicals were acquired from Sigma Aldrich and Merck, both located in Darmstadt, Germany.

### 2.2. Experimental Animals

The clinical study involved a collaboration with a Romanian Spotted cattle farm located in Mureș County, Romania. At that time, the farm housed a total of 240 cattle, including both adults and young stock. The study was conducted between February 2021 and October 2021, during which a subset of 50 cows was identified, for which the breeding date and approximate calving date were verified.

The Romanian Spotted cattle breed traces its origins back to 1860, emerging from an extended crossbreeding program involving native cows and Simmental bulls imported from Switzerland, Austria, Germany, the Czech Republic, and Slovakia. The historical regions of Banat, Transylvania, and Bucovina stood out as the prominent provinces renowned for providing favorable breeding conditions. This breed exhibits a remarkable capacity for environmental adaptation, with optimal performance observed in flat and hilly terrains characterized by a continental climate. In the context of a continental climate, elevated temperatures exert a detrimental influence on milk production, whereas cold conditions result in a significant reduction in daily milk yields. Within the context of small-scale semi-intensive farming, cows of this breed demonstrate an impressively extended productive lifespan, commonly completing six lactation cycles. Remarkably, around 6% of cows attain 8–9 lactations by the age of 11–12 years, and even under challenging conditions, productive longevity typically diminishes to 3.5–4.1 lactations [13].

The shelter infrastructure comprises three walls of reinforced concrete enclosing the structure on three sides, with one long side remaining open. It features an asymmetric roof sloping from a height of 3.2 m at the front to 1.6 m at the rear. The floor is concrete, underpinning a bedding system of straw that is refreshed biweekly and fully replaced every 6–7 weeks or as required. Additionally, the shelter includes two 20-square-meter calving pens designed to isolate nearing-term cows, thereby reducing the risk of conflict and injury post-calving.

Nutritionally, the farm sustains the cattle on stockpiled feed year round, excluding green forage from lactating cows’ diets. In winter, Romanian Spotted cows receive hay of specific botanical composition for optimal nutrition, dispensed in 1.20 m cylindrical bales in a feeder with 12 stations, supporting unrestricted feed access. This collective feeding system, however, complicates precise intake monitoring per individual cow.

### 2.3. Experimental Model

The research encompassed a comprehensive analysis involving 50 bovines, systematically executed through a series of sample collections during both the pre- and post-parturition phases, and adhering to the subsequent protocol.

In the antepartum phase, samples were collected at intervals of 4, 3, 2, and 1 week(s) prior to parturition, designated as AP-W4, AP-W3, AP-W2, and AP-W1, respectively.

Sampling was also conducted at the juncture of parturition (P), encompassing a window of ±12 h surrounding the event.

The postpartum phase involved sequential sampling at 1, 2, 3, and 4 weeks subsequent to parturition, identified as PP-W1, PP-W2, PP-W3, and PP-W4, respectively.

Venous blood samples were collected from the coccygeal vein using vacutainers with a clotting agent. After collection, the samples were left at room temperature to allow for serum separation. The serum was then carefully transferred into Eppendorf tubes, with each tube receiving exactly 0.5 mL. The serum samples were promptly stored at −20 °C in a cryogenic storage facility. The fetal segment of the placenta, identifiable by its cotyledons, was gathered post-partum, preserved in aseptic refrigerated containers equipped with sealing lids, and within 2 h, was subjected to cryopreservation at −80 °C.

**Figure 1:**
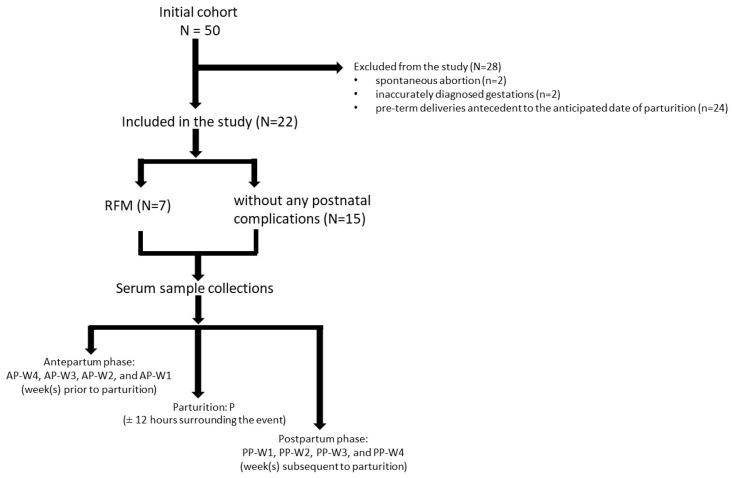
Experimental Model.

Of the initial cohort of 50 bovines, a subset of 22 was deemed suitable for inclusion in the investigative study based on the criterion of permitting the requisite nine sample collections for comprehensive analysis. The experimental phase was characterized by instances of spontaneous abortion (n = 2), inaccurately diagnosed gestations (n = 2), and pre-term deliveries antecedent to the anticipated date of parturition (n = 24). At the end of the study, out of the 50 cows included, 28 were excluded based on the previously mentioned criteria, 7 experienced retentions of fetal membranes, and the remaining cows (n = 15) went through normal birthing processes without any postnatal complications (Figure 1).

### 2.4. Biochemical Analysis

#### 2.4.1. Markers of Oxidative Stress in Serum

Various parameters, including CAT, SOD, MDA, and TAC, were quantified in the serum samples employing specific assay kits. All biochemical analyses were performed using the microplate spectrophotometer SPECTROstar^®^ Nano (BMG Labtech, Offenburg, Germany).

The total SOD activity was measured using a colorimetric assay kit. In this assay, superoxide anions produced by a xanthine and xanthine oxidase reaction oxidize hydroxylamine to form nitrite, which turns purple when reacted with a developer. SOD inhibits this reaction, reducing nitrite formation. One SOD activity unit (U) is defined as the amount of SOD that achieves 50% inhibition in 1 mL of reaction solution. The results were calculated using the kit’s formula and expressed in U/mL. Catalase (CAT) activity was measured by stopping the reaction with ammonium molybdate, which reacts with residual H_2_O_2_ to form a yellowish complex. The intensity of the yellow color, measured at 405 nm, reflects CAT activity. One unit of CAT is defined as the amount of enzyme in 1 mL of serum that decomposes 1 μmol of H_2_O_2_ per minute at 37 °C. The assay protocol was conducted in accordance with the kit’s instructions. The outcomes were expressed as U/mL.

The total antioxidant capacity (TAC) was assessed by employing a commercially available colorimetric kit. The kit assesses the ability of small-molecule antioxidants and antioxidant enzymes to reduce ferric ions (Fe^3+^) to ferrous ions (Fe^2+^), which then form stable complexes with phenanthroline. The total antioxidant capacity is calculated by measuring the absorbance at 520 nm. At 37 °C, a unit of TAC is defined as the amount of sample that increases the absorbance of the reaction system by 0.01 per minute per mL. The assay protocol was conducted in accordance with the kit’s instructions, and the results were expressed as U/mL.

The concentration of malondialdehyde (MDA) was measured using an assay kit, based on the interaction of MDA with thiobarbituric acid (TBA), which results in the creation of an MDA-TBA complex that can be quantified spectrophotometrically. The preparation of the samples followed the kit’s instructions, and the results were given in nmoL/mL.

#### 2.4.2. Tissue Analysis

After removing excess blood by washing and weighing, the placenta (fetal part) samples were homogenized at a tissue–buffer ratio of 1 g/9 mL in PBS (0.01 mM, pH 7.34). After centrifuging the homogenates for two minutes at 10 × 10,000 rpm, the supernatant was utilized for additional analysis.

The Biuret method was used to determine the total protein concentration after collecting the tissue homogenates [14]. The SPECTROstar^®^ Nano (BMG Labtech) spectrophotometer was used to measure the absorbance at 555 nm. The mg protein/g tissue values were given.

Using the same analysis kits, previously described in serum analyses, the following biomarkers were measured from placenta homogenates: CAT, total SOD, MDA, and TAC. All biochemical analyses were performed using the microplate spectrophotometer SPECTROstar^®^ Nano (BMG Labtech, Offenburg, Germany). The results were displayed in U/mg proteins.

#### 2.4.3. Total Lipid Extraction and Determination of the Fatty Acid Profile in Placenta

The extraction of total lipids was performed using a 2:1 mixture of chloroform–methanol. The profile of the different classes of fatty acids (saturated fatty acids (SFAs), unsaturated fatty acids (UFAs), monounsaturated fatty acid (MUFAs), and polyunsaturated fatty acids (PUFAs) was analyzed in the total lipid extracts. The production of the fatty acid methyl esters (FAMEs) from the lipids was achieved by acid-catalyzed transesterification using 1% sulfuric acid in methanol. The FAMEs profile was analyzed using a gas chromatograph (GC) coupled to a mass spectrometer (MS) (Clarus 600 T GC-MS, Perkin Elmer, Waltham, MA, USA). A volume of 0.5 μL of sample was injected into a 60 m × 0.25 mm i.d., 0.25 μm film thickness SUPELCOWAX 10 capillary column (Supelco Inc., part of MilliporeSigma, St. Louis, MO, USA). The operating conditions were as follows: injector temperature 210 °C, helium carrier gas flow rate 0.8 mL/min, split ratio 1:24, oven temperature 140 °C held for 2 min, then increased to 220 °C at 7 °C/min and held for 23 min, electron impact ionization voltage 70 eV, trap current 100 μA, ion source temperature 150 °C, and mass range 22–395 *m*/*z* (0.14 scans/s with an intermediate time of 0.02 s between them). The FAMEs were identified by comparing their retention times with those of known standards in the 37-component FAME Mix (Supelco no. 47885-U) and comparing the resulting mass spectra to those in the NIST MS Search 2.0 database. The amount of each fatty acid was expressed as the percentage of total fatty acid content [15,16].

### 2.5. Statistical Analysis

The statistical analysis was conducted using the GraphPad Prism 9 software program (San Diego, CA, USA). Data were statistically evaluated through an unpaired t test with Welch correction. Significance levels were set at *p* < 0.05, *p* < 0.01, *p* < 0.001, and *p* < 0.0001 to assess the differences between the cows with retained fetal membranes and the ones with normal parturition. All determinations were carried out using the two-stage step-up method (Benjamini, Krieger, and Yekutieli), and the results were presented as the mean values ± standard deviations.

## 3. Results

### 3.1. Antioxidant Enzyme Activity and MDA Levels in Serum

Our study began by examining the SOD activity, a key player in cellular defense against oxidative damage. Our results showed no significant differences in early pre-parturition weeks (AP-W4 and AP-W3), indicating that the OS levels between cows with RFM and those with normal parturition were comparable at this stage. However, a marked increase at AP-W2 (*p* < 0.001, RFM 404.601 ± 20.941 vs. NP 339.101 ± 44.911) suggests a ramp-up in antioxidant defenses or heightened OS as the cows prepared for parturition.

**Figure 2:**
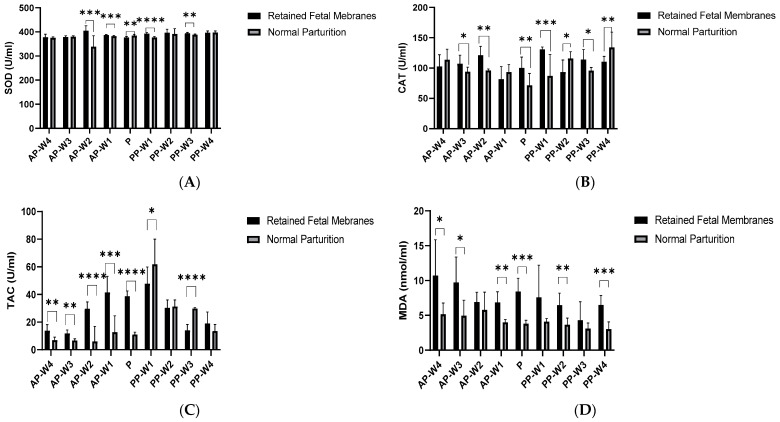
Oxidative stress indicators from serum. (**A**) Superoxide dismutase (SOD). (**B**) Catalase (CAT). (**C**) Total antioxidant capacity (TAC). (**D**) MDA. The outcomes represent mean ± SD of three replicate assessments. * *p* < 0.05, ** *p* < 0.01, *** *p* < 0.001, and **** *p* < 0.0001.

During the act of parturition (P), a significant decrease in SOD activity in cows with RFM (*p* < 0.01, 377.234 ± 4.037 vs. 384.925 ± 5.260) could indicate a depletion of antioxidant defenses under the strain of labor. Postpartum, the initial significant increase in SOD activity at PP-W1 (*p* < 0.0001, RFM 392.600 ± 5.280 vs. NP 377.331 ± 3.583) hints at an acute OS response to RFM (Figure 2A).

Catalase, a pivotal antioxidant enzyme, showed a variable pattern of activity, indicating fluctuations in combating OS. Notably, two weeks before parturition (AP-W2), we observed a significant upsurge in its activity (*p* < 0.01, RFM 121.132 ± 14.831 vs. NP 96.070 ± 2.397), suggesting an increase in hydrogen peroxide detoxification, possibly due to elevated OS in cows with retained fetal membranes. The moderate significance noted at AP-W3 (*p* < 0.05, RFM 107.157 ± 14.064 vs. NP 93.708 ± 7.764) indicates an escalation of this stress as parturition nears. Interestingly, at AP-W4 and AP-W1, the catalase activity did not differ significantly, pointing to critical temporal OS changes tightly linked to the immediate pre-parturition phase.

During parturition (P), the high significance in Catalase activity (*p* < 0.01, RFM 100.193 ± 17.974 vs. NP 71.348 ± 19.770) corroborates with the known physiological stress of birth, particularly intensified in the presence of complications such as RFM. Postpartum, the very significant difference at PP-W1 (*p* < 0.001, RFM 130.650 ± 4.085 vs. NP 87.013 ± 35.486) may reflect the ongoing oxidative aftermath in cows with RFM, affecting their recovery and possibly influencing lactation performance. The later weeks postpartum, especially PP-W4 (*p* < 0.01, RFM 110.454 ± 8.776 vs. NP 133.987 ± 25.242), suggested prolonged oxidative implications, potentially impacting long-term health and productivity (Figure 2B).

Total antioxidant capacity (TAC) provided a broader view of the systemic antioxidant defenses. A significant rise in TAC in cows with RFM during AP-W4 (*p* < 0.01, 13.796 ± 4.421 vs. 6.948 ± 2.142) implies an early compensatory increase in the antioxidant system, possibly in reaction to initial stress or as a preparatory response for the forthcoming parturition stress. The continued significant elevation of TAC through AP-W3 (*p* < 0.01, RFM 11.858 ± 2.456 vs. NP 6.825 ± 1.094) to AP-W1 (*p* < 0.001, RFM 41.563 ± 11.648 vs. NP 12.778 ± 11.792) highlights a sustained antioxidant response leading up to the delivery.

The peak in TAC observed during parturition (P) for cows with RFM (*p* < 0.0001, 38.780 ± 3.727 vs. 11.150 ± 1.555) aligns with the acute oxidative challenges of labor. This suggests that cows with RFM may be experiencing a heightened state of OS or an intensified activation of their antioxidant systems during this critical period. The postpartum phase showed a transient lack of significant TAC difference at PP-W1 (*p* < 0.05, RFM 47.889 ± 11.949 vs. NP 61.873 ± 18.137), which might indicate a brief period when the OS levels begin to wane. However, the significant difference that re-emerges at PP-W3 (*p* < 0.0001, RFM 14.148 ± 4.143 vs. NP 29.896 ± 0.570) and the absence of significant differences by PP-W4 (*p* > 0.05) depict a complex recovery pattern, where OS may fluctuate before reaching a new equilibrium (Figure 2C).

MDA levels provided a direct measure of lipid peroxidation. An increasing trend in MDA, approaching significance at AP-W4 (*p* < 0.05, RFM 10.719 ± 5.137 vs. NP 5.166 ± 1.609), suggested a possible rise in OS as parturition approached, though not reaching conventional levels of statistical significance. The significant rise in MDA levels by AP-W3 (*p* < 0.05, RFM 9.723 ± 3.656 vs. NP 4.959 ± 2.211) highlights a period that is potentially critical in the development of RFM and is marked by increased lipid peroxidation. The significant elevation just before parturition at AP-W1 (*p* < 0.01, RFM 6.876 ± 1.523 vs. NP 4.009 ± 0.395) and the highly significant levels during parturition (P, *p* < 0.001, RFM 8.424 ± 1.894 vs. NP 3.807 ± 0.484) support the hypothesis that OS peaks with labor and may contribute to the pathophysiology of RFM. In the postpartum period, the lack of significant differences in MDA levels at PP-W1 (*p* > 0.05) may suggest an initial decline in OS. However, the significant rise at PP-W2 (*p* < 0.01, RFM 6.479 ± 1.718 vs. NP 3.656 ± 0.954) indicates a potential resurgence or persistence of OS. By PP-W4, the highly significant increase in MDA levels (*p* < 0.001, RFM 6.489 ± 1.350 vs. NP 3.049 ± 1.016) raises concerns about ongoing oxidative challenges or a protracted recovery from parturition stress in cows with RFM (Figure 2D).

### 3.2. Antioxidant Enzyme Activity, Total Antioxidant Capacity, and MDA Levels in Fetal Placenta

**Figure 3:**
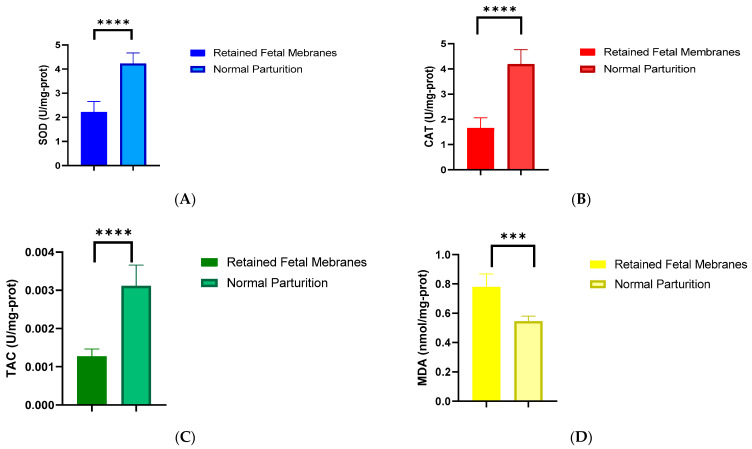
Oxidative stress indicators in fetal placenta. (**A**) Superoxide dismutase (SOD). (**B**) Catalase (CAT). (**C**) Total antioxidant capacity (TAC). (**D**) MDA. The outcomes represent mean ± SD of three replicate assessments. *** *p* < 0.001 and **** *p* < 0.0001.

The decreased SOD and CAT activities in RFM cows (*p* < 0.0001, 2.214 ± 0.441 vs. 4.225 ± 0.438; *p* < 0.0001, 1.651 ± 0.408 vs. 4.191 ± 0.569) are critical findings. SOD and CAT are essential enzymes in the mitigation of oxidative damage, particularly during the stress of parturition. Their reduced activities indicate a compromised capacity to neutralize harmful reactive oxygen species, leading to cellular damage. This impairment could be a contributing factor in the pathogenesis of RFM, as effective management of OS is crucial for normal placental function and the timely release of fetal membranes (Figure 3A,B).

The marked decrease in TAC (*p* < 0.0001, 0.001 ± 0.0001 vs. 0.002 ± 0.0005) in the RFM group further reinforces the notion of an inadequate antioxidant response. TAC reflects the cumulative action of various antioxidants, and its reduction suggests an overall weakened defense against the oxidative challenges of parturition. This diminished capacity to handle OS may exacerbate cellular damage and inflammation, complicating normal placental detachment and contributing to RFM (Figure 3C).

Our findings of elevated MDA levels (*p* < 0.001, 0.779 ± 0.089 vs. 0.545 ± 0.035), a marker of lipid peroxidation, in RFM cows are particularly telling. This indicates increased oxidative damage at the cellular level, which could compromise the structural integrity of the placenta, affecting its function during parturition. The high level of lipid peroxidation may be a direct consequence of the reduced antioxidant enzyme activities and TAC, culminating in the pathophysiological processes that lead to RFM. Elevated MDA levels in the placenta are a clear indicator of OS and lipid peroxidation, both of which are linked to the occurrence of retained fetal membranes in cows (Figure 3D).

### 3.3. The Determination of the Fatty Acid Profile in Fetal Placenta

**Figure 4:**
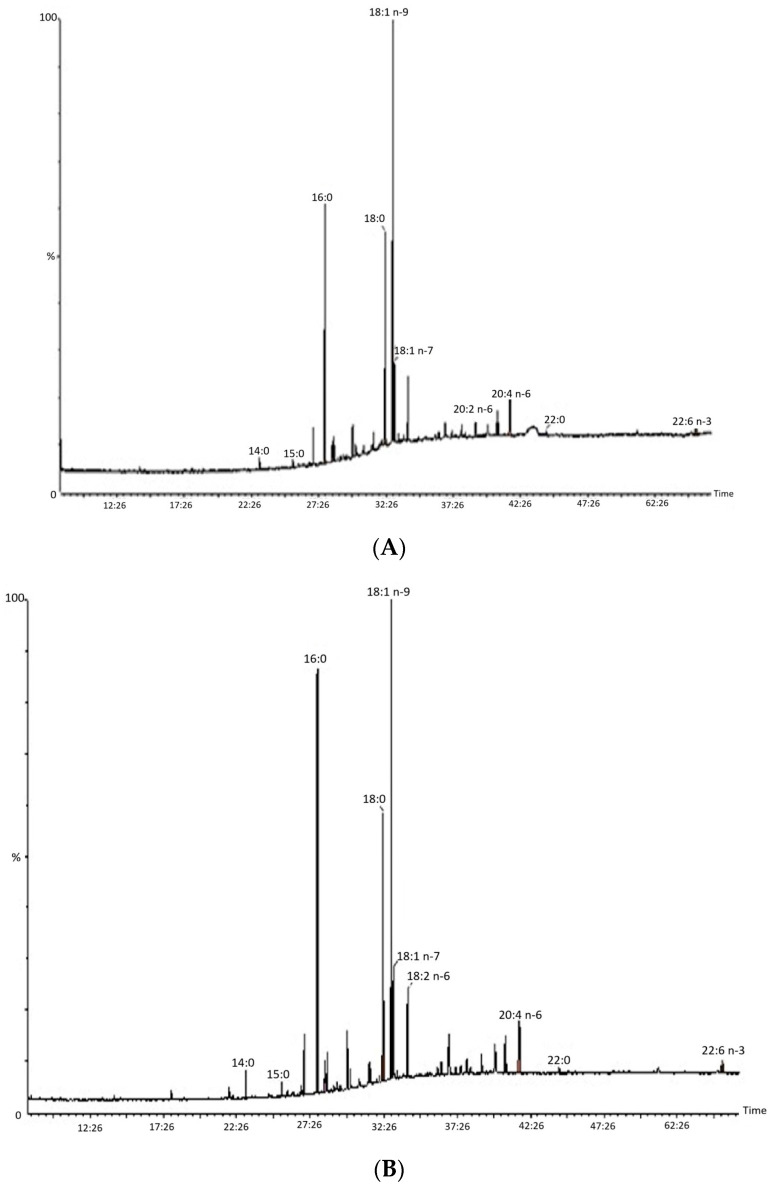
GC-MS chromatogram of the FAMEs from total lipids of the fetal placenta from NP group (**A**) and RFM group (**B**) analyzed with a BPx-70 column. Peaks of fatty acids: myristic (14:0); palmitic (16:0); palmitoleic (16:1 n–9); palmitoleic (16:1 n–7); stearic (18:0); oleic (18:1 n–9); vaccenic (18:1 n–7); linoleic (18:2 n–6); eicosadienoic (20:2 n–6); arachidonic (20:4 n–6), and docosahexanoic (22:6 n–3).

In this study, a total of 11 fatty acids from different categories—saturated, monounsaturated, and polyunsaturated—were separated from the extracts obtained from fetal membranes. Two of the chromatograms obtained are presented in the following figure (Figure 4). Additionally, the results from the quantitative analysis are presented in detail in Table 1.

Table 2 presents a statistical analysis of the results obtained, specifically displaying the fatty acid profiles by category for the two experimental groups.

## 4. Discussion

These results are in accordance with previous studies that have reported an increase in OS and antioxidant activity during the periparturient period in dairy cows [17,18,19]. Pregnancy involves a heightened energy demand for various bodily functions and a greater need for oxygen, leading to increased production of reactive oxygen species [20]. Many genetic, physiological, and environmental factors can impair the cows’ defense mechanisms during the transition period [21], and also, numerous stressors are present around calving, such as parturition, the onset of lactation, and changes in feeding and management practices [22]. During the periparturient period, dairy cattle can face increased disease risk due to the metabolic adaptations caused by the onset of lactation [23]. The metabolic demands experienced by cows during the peripartal period lead to OS, which is caused by the excessive production of reactive oxygen species [20]. Oxidative stress is the main cause of immunological and inflammatory dysfunction in dairy cows in times of high metabolic activity [18], and parturition-related physiological changes correlate with reduced antioxidant defense in cows [24].

In the study conducted by Yazlik et al. [24], the serum level of SOD during the prepartum period was higher in Brown Swiss cows with RFM compared to healthy cows (30 ± 4 U/mL vs. 17 ± 2 U/mL), and no difference was observed in the postpartum period. The activities of SOD were nearly similar in the plasma of both groups of Holstein X Sahiwal cross-breed dairy cows during the transition period in the study by Sharma et al. [25]. Meanwhile, Khudhair et al. [26] demonstrated that SOD activity was higher in serum before calving (6.90 ± 0.18 U/L), and this value was reduced in cows that experienced retained placenta (3.91 ± 0.17 U/L), as well as in healthy cows after calving (4.68 ± 0.36 U/L). However, Li et al. [9] did not observe significant changes between Holstein cows with retained placenta and healthy ones from 21 days before parturition to calving. Still, there were fluctuations in the SOD plasma levels from calving to 21 days postpartum.

In the study conducted by Khudhair et al. [26], CAT activity exhibited a significant increase in cows with retained placenta (11.75 ± 0.37 U/L) compared to cows that experienced normal calving (8.71 ± 0.61 U/L). However, prior to calving, CAT showed reduced activity in cows with retained placentas (5.60 ± 0.31 U/L) in contrast to healthy ones.

Kankoffer et al. [27] conducted an experiment that revealed a pattern in the TAC levels in the plasma of German Black Pied cows with RFM and without RFM during the periparturient period. Analyzing the interaction between placental retention over time and TAC levels indicated that there was an increase in the average TAC concentration between 2 weeks antepartum and 5 days antepartum in cows without placental retention, followed by a decrease at calving. In contrast, in cows with placental retention, the average TAC concentration remained relatively constant at a lower level. Additionally, among cows without placental retention, there was an increase in the average TAC concentration from calving to 1 week postpartum, whereas in cows with placental retention, this increase occurred later, at 3 weeks postpartum. Following this temporary increase in the average TAC concentration, the levels decreased again in both groups. The different timing patterns of TAC in cows with and without RFM might indicate a higher antioxidant requirement in cows with RFM to combat OS in the placenta, which could potentially impact the proper release of the fetal membranes.

Oxidative stress can lead to lipid peroxidation, which acts as a biomarker that helps to identify disease [28]. Due to their low initiation energy and the presence of unsaturated bonds, lipids are susceptible to peroxidative damage [29]. Examining lipid peroxidation byproducts can evaluate the extent of OS [30].

Previous studies have reported an increased quantity of MDA in the serum of buffaloes with fetal membrane retention compared to healthy ones [31]. Li et al. [9] did not observe significant changes between cows with retained placenta and healthy ones from 21 days before parturition to calving. However, from calving to 21 days postpartum, MDA levels showed fluctuations. The MDA level at 7 days after calving was lower in cows with normal calving compared to those with placental retention. A significant positive relationship between MDA and catalase was found in cow’s serum in advanced gestation, whereas non-significant negative correlations were observed in early lactating cows [26].

Lipid peroxidation, as indicated by the plasma MDA concentration, was significantly higher in early lactating cows compared to cows in advanced gestation [26]. Ahmed et al. [32] reported the association of RFM with OS because of increased blood serum MDA, in addition to decreased CAT and SOD activity in Egyptian Buffaloes. The same was reported by Hassan et al. [33], who reported that MDA serum levels were higher in Egyptian buffalos with RFM than in healthy ones while SOD and TAC were increased in those without RFM.

In the fetal placenta, the reduced activities of SOD and CAT, coupled with lower TAC levels in cows with RFM, provide crucial insights into the OS experienced at the placental level. These findings suggest a compromised antioxidative defense, which could impair the placenta’s ability to manage ROS, leading to cellular damage and contributing to the retention of fetal membranes. The elevated MDA levels in the placenta further corroborate this notion, indicating significant lipid peroxidation and potential structural damage that could affect placental function during and after parturition.

It is well-established that placental retention leads to OS within the uterus, resulting in a decreased antioxidant capacity and an increase in reactive oxygen species [27]. This could potentially account for the lower catalase activity observed in the placental tissue of cows with placental retention. However, in contrast, the study conducted by Kankofer [34] discovered that catalase activity in the fetal placental tissue of White–Black breed cows with retained placenta is higher.

This observation is supported by Kankofer [35], who reported that fetal placental SOD activity is higher in White–Black breed cows with retained placenta. Also, in this study, Kankofer et al. [35], presented that the fetal placental SOD activity in White–Black breed cows with retained placenta is higher than in healthy cows (3.03 ± 0.42 U/prot vs. 5.48 ± 0.27 U/prot), which is in contrast to our findings.

SOD activity can influence CAT activity because SOD’s substrate can inhibit CAT and vice versa [36], as cited by Kankofer [35], potentially resulting in uncontrolled reactive oxygen species and ineffective antioxidant defenses in animal tissues [37], as cited by Kankofer [35].

In the study by Kankofer et al. [38], the fetal part in healthy Holstein cows exhibited a lower total antioxidant capacity (TAC) compared to cows with placental retention, while in another study [39], TAC levels were higher in the RFM group (27.90 ± 1.23 vs. 43.50 ± 4.61). Kankofer [40] demonstrated that the concentration of lipid peroxidation products in the maternal and fetal parts of the placenta was higher in animals with RFM compared to healthy animals, as measured immediately after parturition, leading to the conclusion that OS occurs in the placental tissues of cows with RFM [28].

The fatty acid profile of a cow placenta affected by RFM can differ from that of a normally expelled placenta. Typically, the fatty acid profile of a normal placenta includes a balanced composition of saturated, monounsaturated, and polyunsaturated fatty acids. In RFM placentas, some alterations in this profile occur, which may reflect metabolic imbalances, deficiencies, or excesses in certain fatty acids.

Elevated levels of SFAs might indicate increased metabolic stress or inflammation in cows with RFM. SFAs are more stable and less prone to oxidation than unsaturated fatty acids. However, excessive SFAs can be associated with inflammation and metabolic stress, potentially leading to increased MDA levels. MUFAs are less susceptible to lipid peroxidation compared to PUFAs. Adequate levels of MUFAs can help maintain membrane fluidity and reduce OS. Altered MUFA levels can affect cell membrane fluidity and signaling, potentially impacting placental expulsion. PUFAs are essential for anti-inflammatory and immune functions; deviations in PUFA levels can indicate deficiencies or imbalances that might contribute to RFM.

Spectrophotometric methods for measuring lipid peroxidation are frequently used because of their low cost and reproducibility. The oldest and most often utilized methods among these are the conjugated diene determination and the MDA determination. On the other hand, they face criticism for lacking sufficient accuracy. It has been demonstrated that an antioxidant that is non-specific to cells, one that serves to prevent artificial lipoperoxidation during the analysis, must be present for measuring the MDA concentration by a reaction with thiobarbituric acid. Biological samples are susceptible to artificial peroxidation under extreme analytical conditions (high temperatures or an acidic pH) in an aerobic state. Determining the content in UFAs and the ratio between ΣUFA: ΣSFA is an alternative method for determining the level of lipid oxidation [41].

The fatty acid profile of the placenta plays a key role in various physiological and developmental processes in cows. Saturated fatty acids (SFAs), like palmitic (C16:0) and stearic acid (C18:0), contribute to energy storage and cell membrane structure. Monounsaturated fatty acids (MUFAs), such as oleic acid (C18:1), reduce inflammation and promote heart health, while palmitoleic acid (C16:1) has antimicrobial properties and regulates lipid metabolism. Polyunsaturated fatty acids (PUFAs) are essential for fetal brain development and cell membrane integrity. Linoleic acid (C18:2 n−6) is a precursor to arachidonic acid, which is key in inflammation and eicosanoid production, while EPA (C20:5 n−3) and DHA (C22:6 n−3) support neural development and have anti-inflammatory effects [42,43,44,45].

Investigating the fatty acid profile and malondialdehyde (MDA) levels in the placenta can provide insights into the biochemical and OS factors associated with RFM. Changes in the fatty acid profile, especially reductions in MUFAs and PUFAs like eicosapentaenoic acid and docosahexaenoic acid, are correlated with increased OS and MDA levels. Thus, in the case of the experiment carried out by us, the results obtained in the analysis of the MDA concentration are directly correlated with those obtained for the determination of the ΣUFA: ΣSFA.

By analyzing OS markers, such as malondialdehyde (MDA), alongside the fatty acid composition of fetal membranes, we aimed to elucidate the biochemical mechanisms underlying RFM. Our findings indicate a pronounced relationship between elevated OS levels and altered fatty acid profiles in cows affected by RFM, suggesting that these factors contribute to the pathogenesis of the condition. Implementing dietary strategies that balance the fatty acid composition in cattle diets can potentially mitigate OS and improve reproductive outcomes. Supplementing diets with antioxidants and essential fatty acids may foster better fetal membrane health and reduce the incidence of RFM across diverse herds.

Factors such as heat stress, poor housing conditions, and exposure to pathogens can exacerbate OS. These stressors can stimulate inflammatory responses and increase ROS production, further compromising the animal’s health. The mechanisms by which OS contributes to RFM involve several interconnected processes. ROS can initiate lipid peroxidation, leading to the degradation of cellular membranes in fetal membranes. This damage compromises the structural integrity and functionality of the placenta, potentially resulting in the retention of fetal membranes post-partum. Oxidative stress can activate inflammatory pathways, leading to the release of pro-inflammatory cytokines. This heightened inflammatory state can impair normal uterine contractions and delay the expulsion of fetal membranes.

This study has several limitations that should be acknowledged. First, the small sample size limited the statistical power of the analyses, increasing the likelihood of errors. The implementation of exclusion criteria further reduced the number of cows that could be included in the study. Moreover, comparing our results with those from other studies is complicated by variations in breed, feeding practices, and the sources of examined parameters (e.g., plasma versus serum), as well as differences in the units used for reporting the results. Despite these limitations, the findings highlight the necessity for additional research involving a larger sample size to improve the clinical relevance of these biomarkers for understanding the pathogenesis of retained fetal membranes (RFM) in Romanian Spotted cows. Future studies should focus on addressing these limitations and validating the biomarkers in various contexts to enhance their effectiveness in the management of RFM.5. 

This study has successfully identified several biomarkers of OS, including SOD, catalase, TAC, and MDA, which may serve as potential predictors for the risk of RFM in Romanian Spotted cows. These biomarkers showed significant fluctuations in activity and concentration during the periparturient period, underscoring the crucial role of OS in RFM development. A temporal analysis of these OS markers indicated that the highest risk for oxidative damage occurs around parturition, with distinct patterns observed in cows with RFM. Notably, there was a marked increase in OS markers during the critical prepartum weeks, suggesting that these cows undergo heightened physiological stress, which could predispose them to RFM.

In addition, our examination of the fatty acid profiles of placental tissues from Romanian Spotted cows with RFM revealed a breed-specific alteration, characterized by an increase in saturated fatty acids (SFAs) and a decrease in monounsaturated fatty acids (MUFAs). This shift in the lipid profile may contribute to the OS observed in these cows and could play a role in the retention of fetal membranes. These findings highlight the significant role of OS in the pathogenesis of RFM in Romanian Spotted cows.

Future research should investigate the applicability of these biomarkers across various breeds to evaluate breed-specific risks and develop targeted strategies to enhance antioxidant defenses during the periparturient period. Such strategies could involve nutritional interventions or other management practices tailored to the specific needs of Romanian Spotted cows.

Understanding the biochemical factors contributing to RFM, such as OS and fatty acid profiles, is crucial for improving reproductive health across various cattle breeds. The insights gained from this study can inform management practices that enhance overall herd welfare and productivity. The findings underscore the need for breeding strategies that prioritize reproductive health. By selecting for traits that are associated with lower OS and enhanced metabolic resilience, producers can improve overall herd fertility and reduce RFM incidence. This has the potential to benefit not only Romanian Spotted cows but also other breeds facing similar reproductive challenges. By emphasizing these broader implications, the paper can serve as a valuable resource for cattle producers and veterinarians seeking to enhance reproductive health and optimize herd management practices across various breeds and production systems.

## Figures and Tables

**Table 1:** Fatty acid composition of total lipids determined by GC-MS in the fetal placenta for RFM and NP groups.

Fatty Acids	Fatty Acid % ^a^	
	NP	RFM
**Myristic (14:0)**	0.856 ± 0.15	1.409 ± 0.09 *
**Palmitic (16:0)**	19.162 ± 0.51	23.431 ± 1.1 *
**Palmitoleic (16:1 n–9)**	2.021 ± 0.18	2.156 ± 1.11
**Palmitoleic (16:1 n–7)**	1.785 ± 0.09	2.115 ± 0.13 *
**Stearic (18:0)**	15.639 ± 0.83	15.001 ± 0.66 *
**Oleic (18:1 n–9)**	33.442 ± 1.32	29.121 ± 1.28 *
**Vaccenic (18:1 n–7)**	5.934 ± 0.13	5.860 ± 0.19
**Linoleic (18:2 n–6)**	5.273 ± 0.05	5.262 ± 0.11
**Eicosadienoic (20:2 n–6)**	1.647 ± 0.19	1.405 ± 0.16 *
**Arachidonic (20:4 n–6)**	4.939 ± 0.21	4.814 ± 0.42
**Docosahexanoic (22:6 n–3)**	2.012 ± 0.15	1.742 ± 1.01 *

a—% of total fatty acids. Values are mean ± standard deviation of samples analyzed individually in triplicate, * *p* < 0.05.

**Table 2:** Statistical analysis of the distribution of the main classes of fatty acids for each group.

Group	Fatty Acid % ^a^				
	ΣSFAs	ΣMUFAs	ΣPUFAs	ΣUFAs	ΣUFAs/ΣSFAs
**NP**	38.459 ± 1.19	44.795 ± 1.46	16.745 ± 1.11	61.540 ± 1.01	1.60
**RFM**	42.837 ± 1.25 *	40.538 ± 1.29 *	16.625 ± 1.08	57.163 ± 1.08 *	1.33 *

a—% of total fatty acids; SFAs—saturated fatty acids; MUFAs—monounsaturated fatty acid; PUFAs—polyunsaturated fatty acids; UFAs—unsaturated fatty acids; *—different from NP.

## Data Availability

The data presented in the study are available in the article.
